# Retriever and Pointer: Software to Evaluate Inbreeding and Genetic Management in Captive Populations

**DOI:** 10.3390/ani11051332

**Published:** 2021-05-07

**Authors:** Jack J. Windig, Ina Hulsegge

**Affiliations:** 1 Wageningen Livestock Research, Animal Breeding and Genomics, Wageningen University & Research, P.O. Box 338, 6700 AH Wageningen, The Netherlands; ina.hulsegge@wur.nl; 2 Centre for Genetic Resources The Netherlands, Wageningen University & Research, P.O. Box 16, 6700 AA Wageningen, The Netherlands

**Keywords:** conservation, genetic resources, genetic management, software, inbreeding, animal breeding

## Abstract

**Simple Summary:**

Small populations can suffer from high inbreeding rates and the associated loss of diversity, inbreeding depression, and expression of genetic defects. Two questions are important for population managers in this respect: (1) What is the status of my population regarding inbreeding? and (2) what is the effect of measures to decrease inbreeding rates? The Retriever and Pointer software has been developed to answer these questions. Retriever extracts relevant data and inbreeding levels from studbook data and Pointer simulates populations to indicate what the effect is of different forms of genetic management. This paper describes the software and gives examples of its use.

**Abstract:**

The Retriever and Pointer software has been developed for genetic management of (small) captive populations The Retriever program uses as input pedigree data and extracts data on population structure that determine inbreeding rates such as skewness of sire contributions. Levels and rates of inbreeding and kinship and effective population sizes are determined as well. Data on population structure can be used as input for the Pointer program. This program uses stochastic simulation to evaluate a population and provides expected levels and rates of inbreeding and kinship, and optionally allelic diversity. The user can simulate different options for genetic management such as sire restrictions, restrictions on inbreeding levels, mean kinships and breeding circles. Both Retriever and Pointer can analyze populations with subpopulations and different rates of exchange between them. Although originally devised for dogs, the software can be, and has been, used for any captive population including livestock and zoo populations, and a number of examples are provide The pointer software is also suitable in education where students may generate their own populations and evaluate effects of different population structures and genetic management on genetic diversity. Input is provided via a graphical user interface. The software can be downloaded for free.

## 1. Introduction

Genetic management is needed for small captive populations in order to prevent high inbreeding rates, loss of genetic diversity, excessive genetic drift, and associated inbreeding depression and expression of genetic defects. To set up an efficient policy it is first important to monitor inbreeding and kinship in the current population, plus those factors that contribute to inbreeding rates. Next, based on the results of this monitoring, measures can be taken to prevent high inbreeding rates in the future. There are many measures possible. Broadly speaking these fall into two categories: (1) changing the population size and/or structure and (2) selecting animals for or excluding them from breeding based on their inbreeding or kinship levels. In this paper, we present software that monitors the current population and extracts relevant parameters from pedigree data (Retriever part) and software that, based on these parameters, simulates inbreeding under different genetic management to indicate suitable genetic management policies (Pointer part).

Theoretically the inbreeding rate (ΔF) in a population can be estimated as 1/2N (N = the number of individuals in the population), or with more complex formula that take for example unequal sex ratios, variation in litter size, overlapping generations, selection or presence of subpopulations into account [1]. However, these estimated effective population sizes tend to underestimate the realized effective population size because it is hard to take all relevant factors into account [2], and especially if combinations of different factors play a role formula tend to get complex. For example, for schemes to limit inbreeding rates using exchange between subpopulations, such as a breeding circle, formulas have been derived that can estimate asymptotic inbreeding rates [3] but only for situations where subpopulations have the same size and generations are not overlapping.

Formulas generally estimate what is to be expected on average. However, random events such as a variation in sex ratio of litters may cause higher or lower values than expected, and the variance in inbreeding levels and rates to be expected is of interest as well. Therefore, a better approach can be to estimate inbreeding rates and effective population sizes with computer simulation. Using stochastic simulations, one can in principle input all relevant information and then derive expected inbreeding levels and rates for many years to come. Although it is impossible to mimic the future exactly as it will be, one can determine the range in which future values will lie by repeating simulations including random events with a random number generator.

Our software first uses pedigree data to retrieve information needed for simulating the population, such as number of breeding animals, sex ratios, variation in family size, and on division in subpopulations. It also determines actual inbreeding levels and rates based on pedigree data. There are a number of software programs available that estimate inbreeding levels and rates from pedigrees such as Pedig [4], Endog [5], and CFC [6] but these generally give hardly information on population structure. An exception is the program Poprep [7] which provides extensive tables with information both on population structure and inbreeding and kinship.

The second part of the software simulates inbreeding in populations using data on the population which can be generated by the first part. It simulates a captive population assuming a constant population size, reproduction rate and age structure. This is generally the case in captive populations where owners and population managers determine reproduction. There are many programs that can stochastically simulate genetics of populations including kinship and inbreeding, such as QMSim [8] and SLIM [9]. However, simulating genetic management is generally not possible in these programs. An exception is Vortex software [10] designed for wildlife populations, which offers possibilities for genetic management although not the full range used in practice in captive populations.

Retriever and Pointer software combines the analysis of inbreeding in actual populations with predicting inbreeding and other (genetic) aspects in future generations. Input is via a graphical user interface (GUI) and output is in text files from which graphs may be generated.

## 2. Software

### 2.1. Operation and Graphical User Interface (GUI)

The Windows based program is written in Fortran95 and uses a text file (steering file) with a fixed format as input. For user friendliness, a desktop graphical user interface (GUI) has been designed with the application software PHPDesk (version 47.5) (https://github.com/cztomczak/phpdesktop, accessed on 10 October 2019). The GUI generates the steering file needed for input of the software. The GUI is user friendly and largely self-explanatory although an extensive manual is provided. Moreover, a question mark displayed next to each item providing information when clicked, helping the user to correctly fill in the form. Students are generally able to operate the programs once installed correctly on the computer within minutes, provided that, for example, the pedigree data does not contain errors. There is a separate GUI for the Retriever part and the Pointer part.

After running the actual software, output is generated as a text file which can be accessed and explored via the GUI. Several graphs can be generated, within the GUI. For generating these graphs, the HighCharts JS visualization library (version 2.1.9) (http://www.highcharts.com, accessed on 11 November 2011), a free for non-commercial use charting library written in JavaScript is used. Alternatively, one can copy results from the text file into another program such as a spread sheet to generate one’s own graphs or for more extensive analyses. Currently, the system is set up for windows-based systems only.

The software is free for non-commercial use and can be downloaded, including manuals, from https://genebankdata.cgn.wur.nl/software/software.html (accessed on 6 May 2021). After downloading it can be installed on any Windows based computer. The software can be operated in English or Dutch. Pointer and Retriever are installed as separate programs, and each can be installed and run on its own.

### 2.2. Retriever Software to Monitor Inbreeding and Population Structure

**Figure 1:**
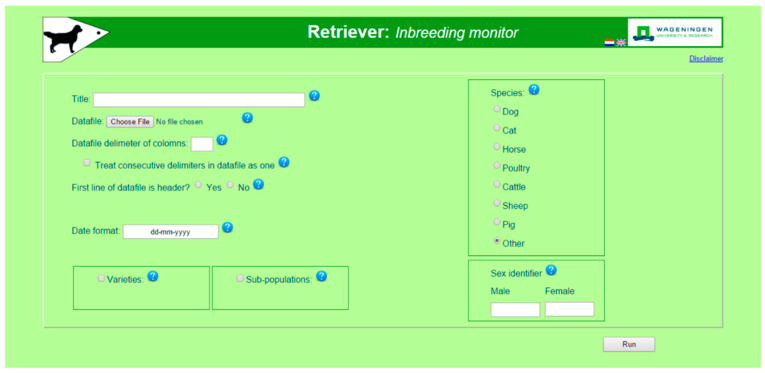
Opening screen of Retriever. Retriever extracts data on population structure that determine inbreeding rates and effective population size plus levels and rates of inbreeding and kinship. Output can be used as input for the pointer program.

The Retriever part of the software analyses pedigree data. Input is given via the opening screen (Figure 1). The main item to be given is the name of the text-file containing the pedigree data. Other information to be provided is how the pedigree data is structured such as content of the columns and how the two sexes are indicated. There is no fixed order of columns and the file may, or may not, contain a header. The user can indicate which species is analyzed, but this has no influence on the actual analyses. The species name is only used in the output, for instance to indicate the offspring of dogs as pups, of horses as foals, etc.

The minimum input required in the pedigree file is the ID of the individuals, their date of birth, their sex, and the ID of their father and mother. Optionally, one can give names of the animals, sub-populations and varieties. Names of the animals are ignored in the analysis, and only used in the output if individual animals are to be indicated, for example when pedigree errors are reported. Both varieties and sub-populations indicate that several groups exist within the population. For every sub-population given, all analyses are repeated and results such as inbreeding rates are reported for the whole population and for each sub-population separately. In addition, average kinships between sub-populations are reported, plus how many animals of one subpopulation are used as a parent in another subpopulation. Under varieties groups can be given for which no separate analyses are needed, and only a table will be provided with the number of animals born each year for each variety. In practice breeds may have different varieties that may be crossed without restrictions (e.g., colour or coat varieties) and for which no information on inbreeding and kinships is needed, but for which studbook keepers may want to monitor frequencies.

Analyses start by checking the data, especially for inconsistencies in the pedigree. When offspring are born before their parents, parents are set to unknown and reported as an error, but analyses continue. Animals used both as a father and a mother are reported as well. When the parents of an animal are not present in the pedigree, a record is created for that parent with its date of birth set to unknown. The pedigree is sorted so that parents occur before offspring. In case sorting cannot take place because of a pedigree loop the analysis stops and an error report is given with individuals present in the pedigree loop.

In total, 17 tables covering aspects relevant for inbreeding monitoring and management are extracted from the pedigree data (Table 1). Tables consist of rows for each year since the first animal was born up to the year in which the last animal was born. For years in between where no animals are born the year is still reported, but generally with values of zero. Animals without known date of birth are ignored in the analyses, and only used for estimating relatedness and inbreeding. At the end, a summary table of data is given that can be used as input for the Pointer software. This summary is based on animals born in the last 6 years of the available pedigree. When other animals are needed to serve as a reference, users need to recalculate the data using the other output tables.

Inbreeding coefficients are calculated after sorting the pedigree using the methods of Meuwissen and Luo [11] and average kinship levels per year using the method of Sargolzai et al. [12]. Inbreeding and kinship rates are calculated over the whole period, and for each 5–6-year period when 10 or more years are present in the data (5 years if the period is a multiple of 5, otherwise excess years are distributed equally over the 5-year periods). Generation based inbreeding rates are calculated for each period of n years using:

$$\Delta F_{1-n}={\left(\frac{1-F_{n}}{1-F_{1}}\right)}^{\frac{L}{n}}$$

where *F*_1_ is the average inbreeding level of pups born in the first year of the period and *F_n_* in the last year and *L* the generation interval. For year based inbreeding rates *L* is replaced by 1. Inbreeding rates are also calculated by regressing LN(1 − F_y_) on year and equating the slope to −∆*F* [13]. Next to inbreeding rates kinship rates (∆f) are calculated using the same procedures but using kinship (f) instead of inbreeding doefficients (*F*). For values above zero realized effective population sizes (Ne) are calculated using Ne = 1/(2∆*F*) and Ne = 1/(2∆f). When ∆*F* or ∆f are 0 or negative the corresponding Ne is set to undefined.

In general, the program can be run on the average lap-top of a student for pedigrees with up to 25,000 individuals within a quarter of an hour. The largest pedigree analyzed with Retriever up to now contained 766,356 individuals spanning 140 years, which took 13 h.

### 2.3. Pointer Software to Simulate Genetic Management

**Figure 2:**
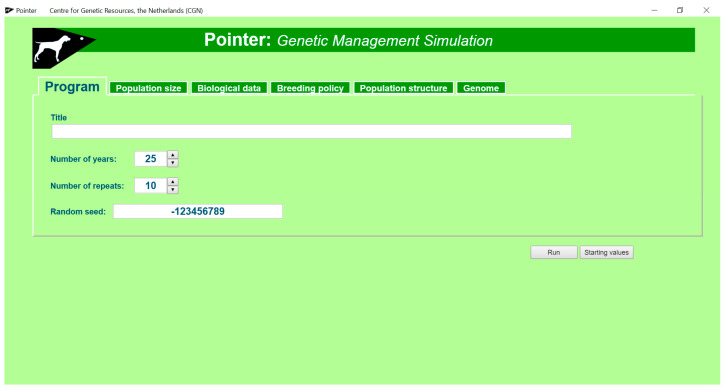
Opening screen of Pointer. Pointer simulates populations with different genetic management to determine inbreeding and kinship rates.

The pointer software predicts inbreeding rates for populations of different size, with different population structure and different genetic management. Populations are simulated in the computer. The minimum input the user has to give is the title, for all other input standard values are available, which the user can change in the GUI. In the opening screen the user can tell the program how many years should be simulated and how often the simulation should be repeated (Figure 2). The program simulates breeding cycles (i.e., periods in which a female can get one litter) and calls these years. If breeding cycles are longer or shorter than a year the user must change ‘year’ in the output to the appropriate length. Repetitions are used to determine possible variation due to random processes, such as the sex of an individual that is born. The program uses a random number generator for random processes. The random seed for the first call to the generator in a simulation can be provided by the user, further calls to the generator use a seed provided by the previous call to the generator.

There are 5 tabs in the opening screen on which the user can click to provide details, of the population (summarized in Table 2) to be simulated. Most details on the population can be derived from studbook data and can be provided by the Retriever part of the software, although the user can fill in any value that seems relevant.

The program uses individual-based stochastic population modelling. It creates a dataset representing the population in the computer, with for each animal its sex, age, sub-population, relatedness with all other animals, and alleles on its genome. Only breeding animals, or future breeding animals, are simulated. When breeding animals are culled their records are deleted and replaced by records of newborn animals. In this way, memory use is limited and the number of years and generations to be simulated not limited by memory constraints.

The program starts with generating the founder population using the number of breeding animals and the percentage of animals in each age class of each sex as provided by the user. These numbers will stay constant during the simulations, generally each culled animal will be replaced by a newborn animal. The assumption is that population managers keep the population constant for example for economic reasons or in order to not overpopulate a farm or a zoo. When not enough animals are born because, for example, of breeding restrictions or genetic defects, population sizes may however decrease. Rather than simulating birth rates and mortality rates generally each year a fixed number of litters is born, and fixed numbers of animals die per age class. In this way captive populations that generally stay constant in number, at least for some period, are simulated. Moreover, it is far easier for users to determine actual numbers per age class of breeding animals rather than to estimate birth and mortality rates.

**Figure 3:**
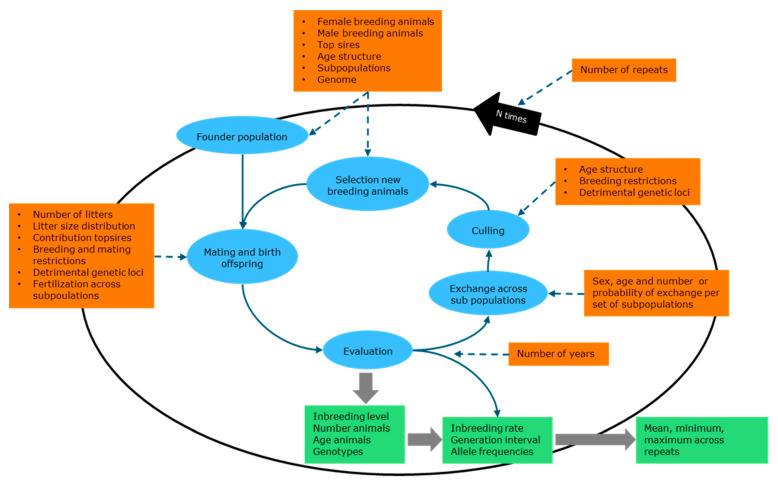
Set up of Pointer software for simulation of genetic management.

After setting up the founder population the program runs a cycle of mating and birth, evaluation, migration across sub-populations, culling and replacing culled animals by young animals born that year (Figure 3). This cycle is repeated for the number of years as given in the input. At the end of the period a new founder population is set up and another round of cycles started, until the number of repetitions as given in the input is reached.

For the founder population individual animal data from an existing population can be used. In this case the founder population is set up as usual. Next, each animal in the computer population is matched to an animal in the real population of the same sex end same age, as far as possible, and receives inbreeding and kinship coefficients of the real animal.

#### 2.3.1. Mating and Birth

For each litter to be born in a year, one of the available breeding females is chosen at random to be the mother. Each female can produce only one litter per year. Generally, the number of litters per year will be smaller than the number of breeding females, given as input by the user, so that not all females will give birth to a litter each year. The birth rate is thus determined by the ratio of the number of breeding females and number of litters born per year. In real life, females not producing in a year can be due to a variety of reasons such as stillbirth, contraception, not being mated, or all offspring being culled or sterilized. Since these causes are generally difficult to determine, the number of litters born per year which can be readily determined in the studbook is used as input. There is, however, the possibility to restrict the use of certain females, for example based on their mean kinships, so that not all females are available for breeding. In these cases, there may be not enough females available for all litters so that less litters are born in that year. Lower fertility of females at later age is accounted for by having a lower percentage of breeding females in later age classes.

For each litter, a male is selected at random from the available breeding males as a father. Males may sire more than one litter in a year. Because of their random choice, the number of litters per sire will approach a Poisson distribution. However, restrictions may apply, such as a maximum relatedness allowed between the father and the mother. Moreover, there is the possibility to indicate popular sires, or dominant males. These males are selected first as father until they have reached their designated contribution, after which the rest of the males are chosen at random for the remaining litters.

To determine litter size the user gives the percentage of litters per size (1, 2, 3, etc., young per litter) as input, which can be determined readily from studbook data as well. The program then draws a litter size according to these percentages. Consequently, variation in offspring per animal is due to being selected as a parent or not, due to the variation in litter size itself and due to variation in lifespan as a breeding animal.

#### 2.3.2. Subpopulations and Migration

The population may exist of several subpopulations each with their own number of breeding males and females but all having the same ratio of breeding females and litters born per year. The standard is that all animals stay within the subpopulation in which they are born, mate with animals in their own subpopulation, and that their offspring remain in their subpopulation as well. However, mating and migration across subpopulations can be specified by the user. For mating across subpopulations input is done by specifying a matrix within each cell either the probability that a litter in subpopulation **a** is sired by subpopulation **b** or the number of litters in subpopulation **a** sired by a father from subpopulation **b** (Table 2). Migration is specified in a similar way: either the probability that an animal from subpopulation **a** migrates to subpopulation **b**, or the number of animals migrating from subpopulation **a** to subpopulation **b**. Migration can be restricted to animals of a certain age or sex. There is the possibility to specify more than one matrix, both for mating across subpopulations and for migration. When more matrices are specified, they are used consecutively over the years.

#### 2.3.3. Culling

When animals age a year the number of breeding animals in each age is adjusted by culling animals or recruiting animals with a more juvenile age so that the original age structure is restored. In this way, a population is simulated where numbers stay constant. When there are less animals than in the previous year the excess is being culled. When there are more animals than in the previous year this would induce a shortage. Therefore, juvenile animals not reproducing are simulated for years that have a lower number of breeding animals than the year class. The number of breeding animals plus juveniles is then equal to the next year so that upon aging enough animals are available. Except for these juveniles only breeding animals are simulated. Culled animals are chosen at random, unless restrictions apply, such as the maximum number of offspring a male is allowed to sire. In real life animals may remain in the population after their reproductive life e.g., after sterilization, at old age or any other cause preventing animals from further reproduction, but in the program, they are removed from the simulation.

#### 2.3.4. Selection of New Breeding Animals

Culled animals are being replaced by animals born in the previous year. Animals are chosen at random from the young born in the previous year in the (sub) population. When not enough animals have been born in that year the population will decrease in size and populations or sub-populations may go extinct. Populations can also go extinct if (too) many restrictions apply for mating or if restrictions are too strict. An example is when only animals are allowed to reproduce that have an inbreeding level below a certain level and as a consequence not enough animals are born.

#### 2.3.5. Genetic Management

Users can specify several options for genetic management. Options can be simulated separately or simultaneously. One class of options are breeding restrictions. These include maximum number of litters allowed per sire per year or per life and maximum number of litters per dam per life. If a restriction per life is reached the animal is removed from the population and replaced by a new-born animal. One can also restrict the number of sons per male that can be selected for breeding. Another class of genetic management is to restrict mating and/or breeding based on kinship or inbreeding levels. One can exclude animals from breeding with inbreeding levels above a threshold or restrict mating to animals with kinships below a threshold. Furthermore, there is an option to minimize the kinship between parents. In this case each female selected for breeding is mated to the male in the population with the lowest kinship with that female. Animals can also be excluded from mating if their mean kinship with all other animals in the population is lower than the average mean kinship in the population. Finally, there is also the option to use optimal contributions to decide which animals are allowed to breed and how many litters each should produce.

Another way of genetic management is to change the population structure. For example, one may enlarge the population or increase exchange between populations. These options can be compared by simulation of populations with different population structures. A special case of genetic management is rotational mating, where the population is split in sub-populations and each sub-population uses sires from a different subpopulation in a systematic way. Rotational mating can be simulated relatively simply in Pointer by specifying the exchange between subpopulations (see Section 3.2).

#### 2.3.6. Genome

Genomic variation can be evaluated for single genes and for strings of up to 32768 loci arranged on one or more chromosomes. Only biallelic loci can be simulated. Initially there is only one set of specifications that applies to all loci so that they are evenly spaced and have identical properties. Special loci that differ in one or more aspects from the other loci can be specified. For each locus, its position on the genome and starting frequency can be specified. Negative effects of loci can be specified as well. For these the selection differential (s) can be specified and the recessiveness (h). The probability of the survival of a homozygous animal for the detrimental allele is 1 − s, where s can vary from 0 (= neutral) to 1 (= lethal). The probability of survival of a heterozygous animal is 1 − hs. If h is 0 the detrimental allele is fully recessive if h = 1 the beneficial allele is recessive. Instead of influencing the probability of survival detrimental loci may also affect fertility.

#### 2.3.7. Output

After running the program, the output screen appears. Several graphs can be chosen, or a text file containing all the results can be opened. This text file can be downloaded, or results may be copied to another program, such as a spreadsheet, for further analysis or to produce different graphs. Output is produced in three sections: general population data, inbreeding and relatedness, and genomic information.

Population size, number of litters born, average age, and extinction of population are produced as general population data. The software is designed to keep population size and age structure constant, so generally there is no variation over the years. However, when not enough animals are born per year to replace culled animals, the population size may reduce and eventually the population may go extinct. This can happen, for example, if not enough litters to be born per year are specified, when genetic defects are too frequent or when genetic management excludes too many animals from breeding. The output serves as a check that population size stays constant and if not, how quickly extinction may occur.

Main output of the software are average inbreeding and kinship values over the years. These are given for all newborn animals and new born animals later selected for breeding, including and excluding self-kinships. Average inbreeding levels are also provided per run (see Section 3.3, below) and per subpopulation. Inbreeding rate is estimated using inbreeding levels in the last year using:

$$\Delta F={\left(1-F_{n}\right)}^{\frac{L}{n}}$$

where *n* is the number of years, *F_n_* is the average inbreeding level in the last year and *L* the average generation interval. Kinship rates are estimated in the same way using the average kinship level (*f_n_*) instead of *F_n_*.

For each locus specified as a special locus, the average allele frequency and genotype frequencies per year are given, for the whole population and separately per subpopulation and per run. For all loci years till fixation or elimination are given as well as the allele frequency in the last year.

## 3. Examples

Three examples are given to demonstrate the operation in practice. The first example is on the sheep breed Blue Texel and Badger Face to show how Retriever works in a population with two subpopulations and the use of Retriever output in the Pointer software. The second example is on rotational mating in zoo populations, to show how the Pointer software can be used to evaluate complex rotational mating designs involving many subpopulations. The final example is the simulation of a dog population used in a student practical to demonstrate the effect of effective population size on genomic variation.

### 3.1. Blue Texel and Badger Face Sheep

The Blue Texel is a local Dutch sheep breed that started as a colour variety of the Texel breed, but is now managed as a separate breed with its own herd book since 2000. Another colour variety, the Badger face, appeared within the Blue Texel and has been registered since 2004 in the same herd book. The breeding organization made the herd book data available for analysis. These consisted of records of 116,878 animals born between 1986 and 2019. The data of animals born before 2001 contained information only of animals that later were used in breeding. Consequently, information on total population size, litter size etc. could only be retrieved for the years 2001–2019. Retriever software was used to analyze the population structure, inbreeding, and relatedness, up to the then current (2019) population. Pointer software was used to determine future inbreeding rates under different scenarios, in particular whether managing the Blue Texel and Badger face as separate breeds can be sustainable.

**Figure 4:**
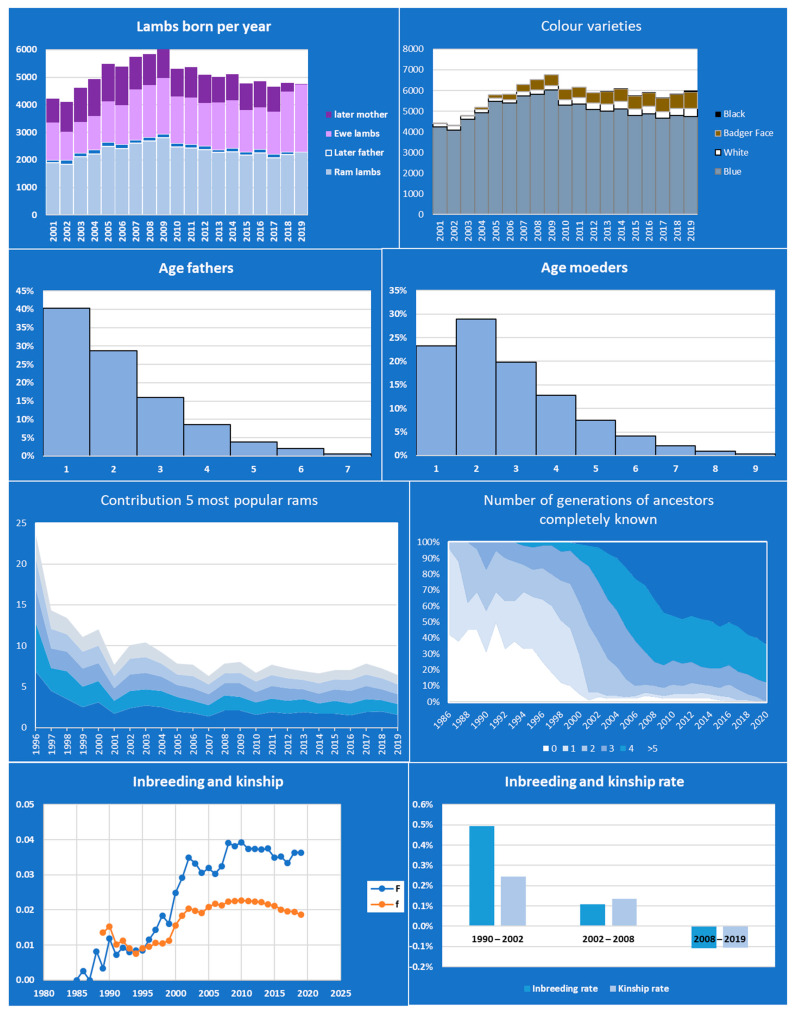
Main results from the Retriever software analysis of the Blue Texel herd book data.

Error reporting by the Retriever software helped to solve a pedigree loop. After the pedigree loop was solved the Retriever software took almost half an hour to analyse the 116,878 animals in the data base. The output text file was imported into a spreadsheet, so that data of years with complete information (2001–2019) could be selected and graphs produced (Figure 4). Main findings are that the population size of the Blue Texel is large enough (4000–6000 lambs born per year) to enable a sustainable genetic management; 9.8% of the male lambs and 48.3% of the mothers are later used for breeding; generations are overlapping and ewes breed up to 9 years old and rams up to 7 years; 52% of litters are twins, 5% triplets; Contributions of rams were skewed before 2000 (up to 24% of lambs sired by only 5 rams) and less skewed afterwards (around 7.5% sired by the 5 most popular rams); pedigree completeness increased, although at a slower rate since 2008; inbreeding and kinship increased but rates never exceeded 0.5% per generation and even decreased somewhat since 2008. Simulations in Pointer confirmed low inbreeding rates for the current population, but excessively high inbreeding rates in case the Badger face variety is managed as a population on its own, without input from the Blue Texel. All in all, the Blue Texel population seems to be a healthy population from a genetic point of view.

### 3.2. Rotational Mating to Manage Groups in Zoo Populations

Zoo populations can be an important reservoir to conserve biodiversity. Zoos can harbor only a limited number of animals and import from the wild is to be avoided. Consequently, the genetic management of zoo populations is needed to reduce the loss of genetic diversity. Pedigree or DNA based breeding programs are used for species in which individual animals can be recognized. For some group living animals, such as fish or deer, it can be impossible to determine parentage of animals and to determine which animals reproduce and form mating pairs. In these cases, alternative forms of genetic management are needed. Rotational mating is one form of genetic management where no pedigree knowledge is needed. We used the pointer software to investigate the effectiveness of rotational mating.

Rotational breeding originated in livestock breeding [14,15] and involves the exchange of individuals between groups in a systematic way. The distinction between groups is maintained while the systematic exchange of animals between groups prevents high inbreeding rates within groups. Animals only breed in a single group during their entire life to maintain genetic diversity between groups. Only newborn animals or young animals not having reproduced yet are exchanged. Inbreeding levels and rates can be derived mathematically for relatively simple situations e.g., no overlapping generations and fully random mating within groups for equally sized sub-populations [3] or for groups of different size [16].

**Figure 5:**
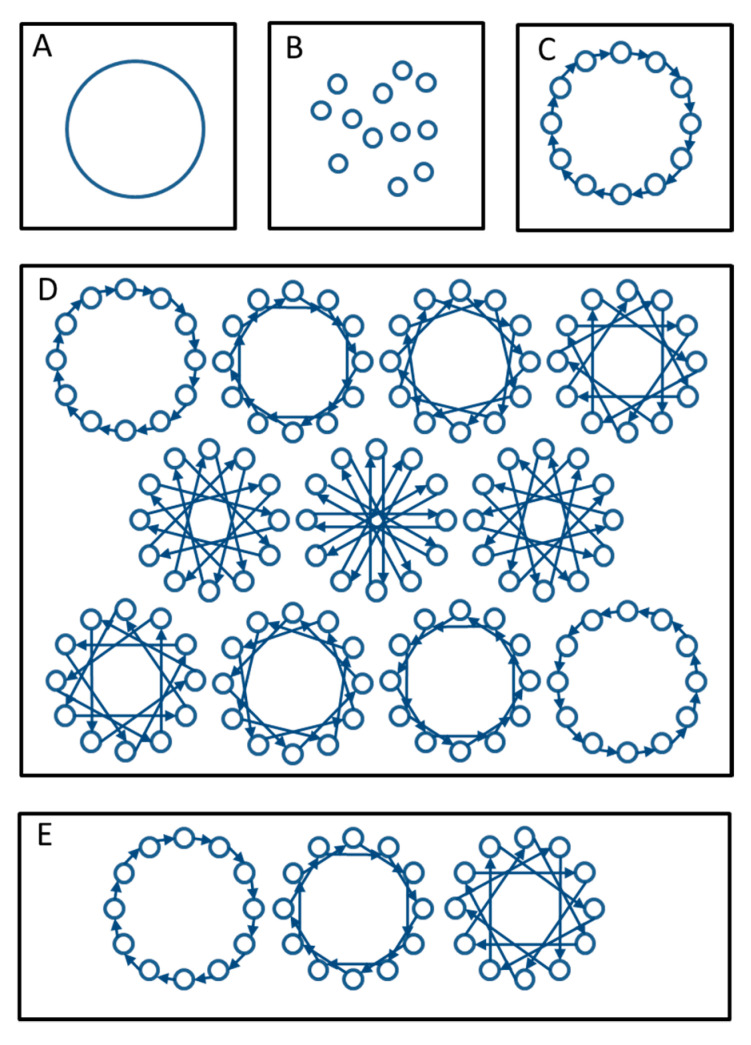
Breeding schemes, involving 12 groups, that were simulated using the Pointer software, including three schemes for rotational mating. Circles represent groups, arrows indicate exchange of animals, pointing from donor to recipient group. (**A**) Maintained as one single large group; (**B**) Isolated groups without exchange; (**C**) Breeding circle; (**D**) Falconer’s scheme; (**E**) Maximum avoidance of inbreeding. (**A**–**C**) each generation same scheme; (**D**) each 12th generation the same scheme; (**E**) each 4th year the same scheme.

There are several types of rotational mating. Breeding can be organized in a breeding circle where donor-recipient groups are always the same. Here, the first group donates animals to the second group, the second to the third etc. and the last group to the first group (Figure 5C). Alternatively, one can change donor-recipient combinations each breeding cycle to avoid mating related animals as much as possible. There are several schemes possible in this respect (for an overview see [3], in practice results are often quite similar for different schemes). Here we will consider two of the more diverging schemes: Falconer’s scheme (Figure 5D) and maximal avoidance of inbreeding (MAI) (Figure 5E) and compare them with a breeding circle. In Falconer’s scheme one starts as in a breeding circle, but each year a donor group is coupled to a different recipient group until all combinations have been used after which a new cycle starts (see Figure 5D for the case of 12 breeding groups). Under MAI one starts as in a breeding circle but in the next years avoids groups that are related through exchange in previous years.

The Pointer software was used to determine inbreeding rate, effective population size and loss of diversity for the different scenarios in a population split into 12 sub-populations (Figure 5). Each sub-population consisted of 2 males and 12 females, populations were overlapping, and litter size varied from 1 to 4 (somewhat comparable to a deer population). Rotational mating schemes could be easily run by specifying that males of age 0 (i.e., newborn males) had a 100% probability of migration to the other sub-population. To specify multiple migration schemes, as used in the simulation of Falconers scheme and for MAI, we made use of the possibility to specify consecutive migration schemes in the software. Inbreeding rates were determined for several periods and compared to mathematically derived rates for simplified scenarios (non-overlapping generations, no variation in litter size). Each scenario was simulated for a period of 1000 years and with 50 replications. The actual running of a scenario and its replications took less than 5 min on the available laptop computer. A spreadsheet program was used afterwards to produce tables and graphs from the text file that was produced as output.

**Figure 6:**
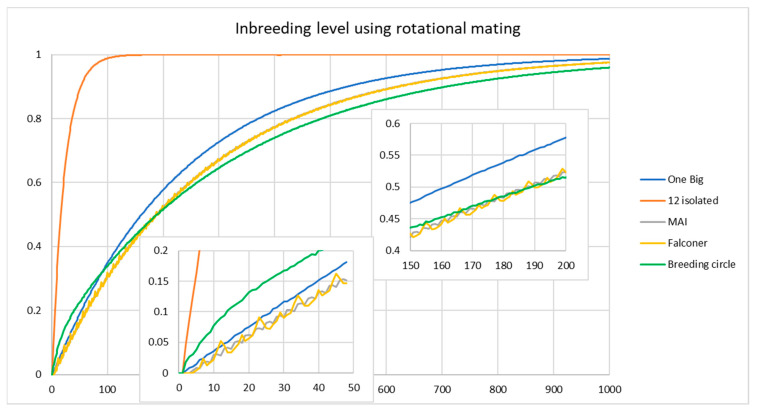
Inbreeding levels for 1 large group consisting of 24 males and 144 females (blue line) compared to 12 groups with 2 males and 12 females each, either without exchange between groups (orange line) or with systematic exchange of all new born males under 3 breeding schemes (details in Figure 5). Inbreeding levels are average of 50 simulation replicates performed with the pointer software.

The results allowed us to distinguish between the short-term and long-term effects of the different scenarios. Which scenarios provided the lowest inbreeding rates differed over the years (Figure 6). Inbreeding rates for populations split in groups and maintained by rotational breeding were always much lower than for isolated groups. The inbreeding level was higher under a breeding circle than for a single large group with random mating for the first 78 years, and higher than Falconers scheme and MAI up to about year 180.

Inbreeding rates determined by the pointer software were slightly higher than those derived mathematically for populations with none overlapping generations. The main reason was that with the simulations not all males were used in each breeding round due to the random choice of males for each mating, while in the mathematical calculations it is assumed that each male contributes to the next generation although fully random mating is assumed. The software was proven to be very efficient and allowed us to generate results within a short time span giving valuable insights in rotational mating. The software has also been used to evaluate the effect of partial breeding circle for Hamadryas baboons in zoos, where instead of transferring all males a limited and fixed number of females was transferred between zoos [17]. Although less efficient than a full breeding circle partial breeding circles could be an efficient way to reduce inbreeding rates and loss of genetic diversity.

### 3.3. Student Practical on Allele Frequencies and Effective Population Size

Effective population size can be a notoriously difficult concept to grasp for students. Although the actual population size does influence the effective population size, in practice uneven contributions of parents to reproduction are more important. Even more difficult to understand is that as a consequence of a small effective population size genetic drift increases and allele frequencies can vary considerably. Simulations can help to better understand this effect by replicating the same scenario many times. We devised an exercise for students where the same population of the Saarloos Wolfdog [18] is simulated twice. Once it is simulated with each sire having an equal chance of reproduction giving a larger effective population size, and once with one dominant sire contributing to 90% of the offspring in the next generation.

After an introduction of the software in a lecture of 15 min, most students were able to operate the program and run the exercise independently. Students had to run 25 replications of both scenarios and plot the allele frequency of a neutral allele starting at a frequency of 0.5 for each separate replication, for 25 years. In 2019, exercises were run in a classroom on university computers where the software was installed in advance. In 2020 and 2021, students had to run the exercise on their own computer at home due to measures restricting the spread of COVID-19. Some students encountered difficulties with fire walls preventing the installation of the software or preventing the software to write output on the computer. Once such problems were solved all students could run the software and output was produced within 10 min.

**Figure 7:**
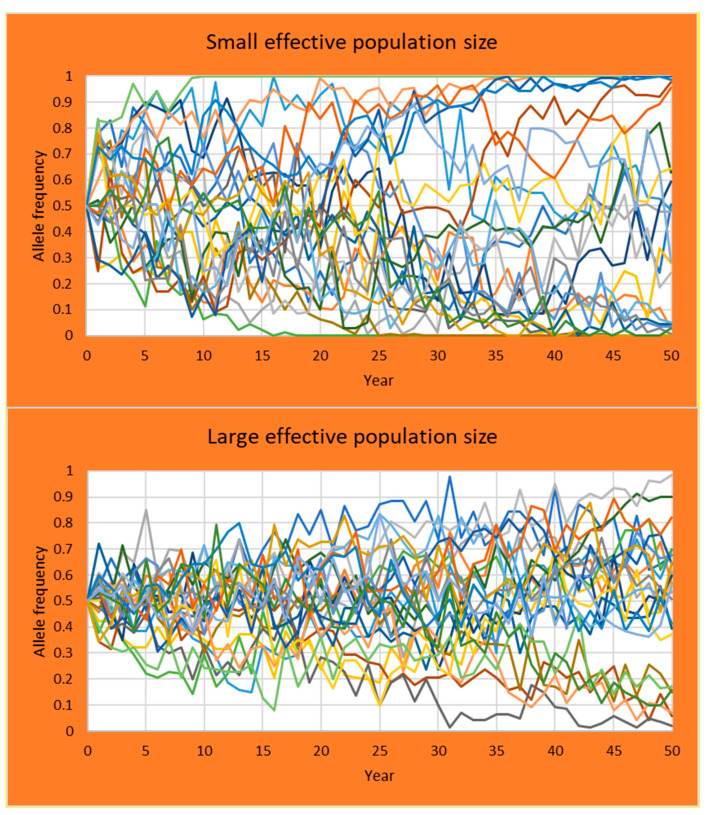
Output of the results for the students practical devised to understand the effect of effective population size on variation in allele frequencies. Same population was simulated twice, once with breeding dominated by a single sire producing a small effective population size (top panel), and once with all sires having an equal chance to reproduce (bottom panel). Shown is the allele frequency in each of the 25 replicates.

Students first calculated effective population size from the census numbers and the inbreeding rates for both scenarios. Next, for each scenario a graph was produced with the simulated allele frequency in each of the 25 replicates (Figure 7). The graphs clearly showed that on average the allele frequency remain at 0.5, but that there is variation across replicates. This variation is considerably larger in the scenario with the single sire dominating reproduction producing a small effective population size.

## 4. Discussion

Monitoring genetic diversity and inbreeding and determining the effect of genetic management are essential for small populations, but is often quite difficult. The software described in this paper is developed to ease that task. The Pointer and Retriever software can support genetic management based on data available for all breeds that keep studbook data. Even when there are no (reliable) studbook data effects of genetic management can still be evaluated by simulation with the Pointer software. The only information needed to run Pointer is the number of breeding animals, litter sizes and age structure. Although specifically designed to evaluate small populations and genetic management, the software proved to be flexible and capable to analyze more complex populations and situations.

For many populations, especially dog populations, the Retriever software enabled us to quickly retrieve an extensive overview of inbreeding and population parameters. Generally, most time was needed for correcting errors in the herd book data such as offspring born before parents. Other mistakes such as 1969 instead of 1996 for the year of birth can produce strange results such as very long generation intervals, and can be hard to recognize and rectify.

Critical reflection on the produced output is always needed. This is especially needed for inbreeding rates. Inbreeding rates may vary over different periods. Therefore, the software estimates inbreeding rates over the whole period, and over fixed 5 years periods, assuming a constant rate and generation interval within the period. Changes in inbreeding rates may follow a different pattern. A visual check of the inbreeding level vs. year graph will help to determine whether this is the case, or whether other periods need to be defined for the calculation of inbreeding rates and associated effective population sizes.

Irregular inbreeding and kinship levels over time can have different causes. Increases in population size, mixing of subpopulations, import of animals from outside the population are some examples, and may even cause a decrease in inbreeding levels and a negative inbreeding rate. Unknown parents for some animals may cause drops in inbreeding levels as well. Note that in the case of negative inbreeding or kinship rates the effective population size loses its meaning and is not defined (compare [19]). The output can help to relate changes in inbreeding levels to various causes such as changes in population size or (absence of) pedigree completeness. Comparing kinship and inbreeding levels will help to determine whether breeding is random or, for example, confined to sub-populations.

The Pointer software enables the evaluation of genetic management in a relatively simple way, contrary to the often quite complex mathematics involved in estimating inbreeding rates for different genetic management. It also helps to demonstrate the effectiveness, or absence of effectiveness, of different genetic management. These can be hotly debated in breeding organizations, while the underlying principles are not easy to explain. For example, the effect of a limit on inbreeding, or a ban on mating animals with common ancestors in the past x generations is often promoted, but not very effective in the long run. The explanation is that inbreeding is not heritable, and kinships may still increase despite these measures causing problems in the longer run. But this explanation can be hard to get across, while a simple graph showing the expected inbreeding rates under different genetic management [20] can be very effective. Similarly, the use of animals from outside a breed (outcross) is often heavily debated. Proponents argue that inbreeding levels will drop considerably and opponents that genetic defects from outside the breed may be introduced. Simulations of outcross, using the option to simulate subpopulations and genetic defects [18] can demonstrate to what extent the arguments hold, and what the effect is of backcrossing after the outcross.

Another example where simulations helped to decide on different management options was the selection for scrapie resistance in sheep in the Netherlands [21,22,23]. Under EU regulations use of rams other than fully resistant for scrapie due to the homozygous ARR/ARR allele of the prion gene was to be banned. Simulations helped policy makers to decide on exemptions for rare breeds and showed that the temporary use of heterozygous ARR rams hardly postponed the increase in ARR levels to sufficient levels but prevented the increase of inbreeding rates to unsustainable levels.

Simulations are a computer model of reality and can only approximate real values. Incorporating the main factors determining inbreeding and kinship levels is of overall importance. We have compared inbreeding rates in real populations of purebred dogs determined with pedigree data (Retriever program) with simulation results of the Pointer program. Results showed a reasonable match between the expected and the real values, and that the real values were always within the predicted range. Most crucial parameter proved to be the contribution of dominant males. Without incorporating the contribution of the top 2 to 5 most popular sires, simulations generally underestimated inbreeding rates.

Over time the software has been developed and adapted to most practical situations. Future extensions will be developed continuously. The examples under study are the simulation of breeding values in Pointer, the definition of a reference population for the summary table in Retriever and founder analyses in Retriever. We are also studying the possibilities to deal with incomplete pedigrees in Retriever, e.g., use of individual inbreeding rates to estimate population inbreeding rates [24,25] and regressing pedigree completeness on year of birth to compare rates of pedigree completeness to the generation interval.

Software to simulate breeding programs and breeding values, such as MoBPS [26] is more suited to evaluate all the complexities of breeding programs and estimate not only the effect on inbreeding levels, but also on genetic change and progress. However, extending our software with the evaluation of breeding values proved to be possible. The effect of selection on breeding values for hip dysplasia in dogs with exchange between subpopulations was successfully evaluated for Labradors [27]. The possibility to evaluate breeding values for polygenic traits will be added to future versions of the Pointer software. Another extension will be to simulate different periods with different parameters. For example, to evaluate what happens if the population size is suddenly reduced. Currently, only differences in the exchange of individuals between subpopulations can be specified for different periods.

## 5. Conclusions

Retriever and Pointer software is relatively simple to use but can evaluate complex populations with different options for genetic management. It can help to make better decisions for breeding organizations and policy makers regarding measures to prevent too high inbreeding rates. It also can help to visually demonstrate effects of different aspects of population structure and genetic management. This can be useful for boards of breeding organizations to convince breeders to adopt certain measures. It can also be useful in education to explain population genetic principles.

## Figures and Tables

**Table 1:** Overview of output tables of the Retriever software.

Item	Description	Period	Columns
Population size	Number young born	Per year	Males, females, total not used and later used in breeding
Parents and offspring per year	Number of parents and offspring/parent per year	Per year	#mothers, #fathers, average litter size, #litter/father, #offspring/father
Offspring/parent per life	Average number of offspring per parent in its future lifetime per year	Per year	Males/Females, Mean, standard deviation, maximum
Litter size	Number of litters of a certain size per year	Per year	Litter size 1, 2, etc.
Age fathers	# of young born with fathers of a certain age	Per year	1, 2, 3 etc. years old
Age mothers	# of young born with mothers of a certain age	Per year	1, 2, 3 etc. years old
Generation interval	Average age of parents at time of birth	Per year	Males, females, parents
Pedigree depth	Generations of ancestors known	Per year	Generation equivalent, % with 0, 1, 2, 3, 4, >4 generations of ancestors completely known
Top-sires	Contribution of top sires to total number of offspring	Per year	# of fathers, contribution of 1st, 2nd,..., 10th most popular sire
Varieties	Number of young born	per year	Variety1, 2 etc.
Inbreeding and kinship	Average coefficients of young	per year	Inbreeding, kinship including and excluding self-kinships, kinship future parents, fathers, mothers
Inbreeding and kinship rates	Delta F	Entire period, + per 5 year	Year based, generation based, effective population size if deltaF > 0
Sub-population numbers	Number of pups born	Per year	Subpopulation 1, 2 etc.
Descent of Sub-populations	Origin and number of parents for pups born in each subpopulation	Entire period	Subpopulation 1, 2 etc.
Sub-populations relatedness	Average relatedness between pups	Per year	For each combination of subpopulations
Summary for Pointer software	Summary of results to be used as input in pointer software	Last 6 years	#litter, breeding males and females per year, contribution top 4 sires, liter size and a parental age distribution

**Table 2:** Overview of input for the Pointer software.

Parameters	Example	Description
number of years number of runs	100, 25	Less years if population goes extinct
Random seed	−123,456,789	Starting value for pseudo random number generator
number of breeding animals	10 males, 50 females	Will stay constant, unless not enough animals are born or genetic management or genetic defects limits numbers
number of litters/year	25	Will stay constant unless not enough parents available
Litter size distribution	0.20 0.70 0.10 for litter size 1,2 and 3	Will stay constant, but see under number of breeding animals
Age distribution	0.75, 0.20 0.05 for Ages 1, 2 and 3	
Number of top sires plus their contribution	4, 0.50	
number of subpoulations and size	2 with sizes 2, 8 (males) and 20, 30 (females)	
Genome data: number of Loci, number of chromosomes, map length, mutation frequency	10, 2, 1 Morgan 1 × 10^−6^	Up to 32,768 Loci can be specified
Loci data: starting frequency, mortality, mortality heterozygote, first year when effective	0.50, 100%, 0%, 0	Can be specified for all loci or individual loci. Effect can be on fertility or survival, selection against carriers of alleles possible
Genetic management: restrictions on number of offspring, relatedness, inbreeding, Mean kinships or use of optimal contributions	5 liters per sire per year	(Combinations of) options can be set on or off
Fertilization across subpopulations	1.0 0.0 0.1 0.9	On diagonal probability (or number) of litters sired by males from own subpopulation, off diagonal by males from other subpopulation. These can be varied between years. Example specifies that females of subpopulation 2 have 10% chance being fertilized by a male from 1st subpopulation
Migration between sub-populations	* 0 5 *	Off diagonal number (or prababilty) of animals migrating between subpopulations. * on diagonal indicates all other animals remain in their own subpopulation. Migration can be restricted to ages or sexes, can be varied over years. Example specifies that each year 5 animals migrate from subpopulation 1 to 2

## Data Availability

Restrictions apply to the availability of data used for the example involving the Blue Texel. Data was obtained from “Stamboek Blauwe Texelaars” and are available from the authors with the permission of “Stamboek Blauwe Texelaars”.
